# Key Microbiota Identification Using Functional Gene Analysis during Pepper (*Piper nigrum* L.) Peeling

**DOI:** 10.1371/journal.pone.0165206

**Published:** 2016-10-21

**Authors:** Jiachao Zhang, Qisong Hu, Chuanbiao Xu, Sixin Liu, Congfa Li

**Affiliations:** 1 College of Food Science and Technology, Hainan University, Haikou, 570228, P. R. China; 2 College of Materials and Chemical Engineering, Hainan University, Haikou, 570228, P. R. China; Dong-A University, REPUBLIC OF KOREA

## Abstract

Pepper pericarp microbiota plays an important role in the pepper peeling process for the production of white pepper. We collected pepper samples at different peeling time points from Hainan Province, China, and used a metagenomic approach to identify changes in the pericarp microbiota based on functional gene analysis. UniFrac distance-based principal coordinates analysis revealed significant changes in the pericarp microbiota structure during peeling, which were attributed to increases in bacteria from the genera *Selenomonas* and *Prevotella*. We identified 28 core operational taxonomic units at each time point, mainly belonging to *Selenomonas*, *Prevotella*, *Megasphaera*, *Anaerovibrio*, and *Clostridium* genera. The results were confirmed by quantitative polymerase chain reaction. At the functional level, we observed significant increases in microbial features related to acetyl xylan esterase and pectinesterase for pericarp degradation during peeling. These findings offer a new insight into biodegradation for pepper peeling and will promote the development of the white pepper industry.

## Introduction

Pepper (*Piper nigrum* L.), one of the most famous spices in the world, is an important member of the family *Piperaceae*. It is native to India, and is mostly cultivated in tropical and subtropical regions [1]. Pepper fruits contain 1.0%–2.5% volatile oil and 5%–9% alkaloids, mainly piperine, chavicine, piperidine, piperetine, and resin [2]. Pepper alkaloids exhibit a wide variety of biological effects, including immunomodulatory, anti-carcinogenic, anti-asthmatic, stimulatory, hepatoprotective, anti-inflammatory, anti-microbial, and anti-ulcer [3, 4]. Pepper is therefore also widely used in medicine and health care [5, 6].

China is one of the largest pepper producers in the world, with an estimated annual production of 27,210 tons. Hainan Province produces more than 90% of the country’s pepper crop, with more than 80% of the product being white pepper [7]. The traditional method for pepper peeling is known as retting. In this process, ripe pepper fruits are separated from the stalk, tightly packed into jute bags, and steeped in still or flowing water for 7–14 days. During this procedure, the pericarp decays, and the pepper is kneaded until the pericarp is removed. The method is still used by most pepper farmers because of its simplicity. However, it is time-consuming and produces an unpleasant skatole smell [8, 9]. It therefore makes sense to improve on the current pepper peeling technology.

In recent years, biodegradation has been considered as a new approach for pepper peeling [10, 11]. Pectinase produced by various pericarp microbes plays a key role in pericarp degradation and peeling. Compared with traditional peeling technology, biodegradation is advantageous in terms of time consumption, product quality, and level of environmental pollution [11]. However, previous research showed that use of a single microbe for peeling resulted in a poor quality of white pepper [12]. Therefore, we suggested that the peeling process required a complex enzyme system produced by various interacting microbes rather than a single microorganism [13, 14]. Accordingly, it is necessary to explore the functional genes of key microbiota to develop the biodegradation method of pepper peeling.

With the development of next-generation sequencing (NGS), the high-throughput sequencing-based metagenomic approach has been widely applied in microbiology [15, 16] to reveal the dynamic changes in the structure of microbiota and their functional genes [17]. In the present study, pepper samples were collected from Hainan Province, China, at different peeling time points. The metagenomic approach was used to explore core microbes and functional genes related to pericarp degradation during pepper peeling.

## Materials and Methods

### Experimental design and pepper sample collection

A longitudinal study design was used to investigate core microbiotas and their functional genes during pepper peeling. Pepper samples were collected in triplicate at the pepper farms in different peeling time points from Qionghai city (Group 1) and Wanning city (Group 2) of Hainan Province, China. When peeling, the pepper fruit (containing pericarp, pulpa and one pepper kernel) was tightly packed into jute bags, and retting in still or flowing retting in the water for 6 days, then the peeled pepper was obtained. Detailed sample information is listed in Table 1. The pepper samples were collected on private pepper farm in Qionghai and Wanning. After obtaining the written informed consent, the owner of pepper farm gave permission to collect pepper samples and conduct the study on this site.

**Table 1:** Sample information and α diversity in present study.

Group	Sample	Time Point	Reads	OTU number	Shannon index
**Group 1 Qionghai City**	Pep3 (*n* = 3)	0 day	19082±1225	126±22	2.55±0.11
	Pep5 (*n* = 3)	1 day	18644±2782	178±15	3.25±0.08
	Pep7 (*n* = 3)	2 day	19725±955	285±38	4.22±0.23
	Pep9 (*n* = 3)	3 day	19921±842	247±17	3.97±0.14
	Pep11 (*n* = 3)	4 day	20251±3046	191±8	3.63±0.21
	Pep13 (*n* = 3)	5 day	19836±1958	201±24	3.71±0.16
	Pep15 (*n* = 3)	6 day	18882±495	199±16	2.42±0.16
**Group 2 Wanning City**	Pep2 (*n* = 3)	0 day	20031±795	135±7	2.68±0.09
	Pep4 (*n* = 3)	1 day	19968±1034	166±14	2.98±0.17
	Pep6 (*n* = 3)	2 day	19845±1684	277±19	4.18±0.26
	Pep8 (*n* = 3)	3 day	18964±2131	235±51	3.86±0.51
	Pep10 (*n* = 3)	4 day	19047±3017	175±21	3.22±0.14
	Pep12 (*n* = 3)	5 day	18995±1320	142±18	2.72±0.27
	Pep14 (*n* = 3)	6 day	19008±982	186±29	2.23±0.13

### Sample processing and DNA extraction

DNA was extracted from pepper samples using a Qiagen DNA Mini Kit (Qiagen, Hilden, Germany) in combination with a bead beating method [18]. Isolated microbial DNA was then used as a template for further sequencing.

### Polymerase chain reaction (PCR) amplification, quantification, pooling, and sequencing

The V3-V4 region of 16S ribosomal RNA (rRNA) genes was amplified as described previously [19]. A set of 6-nucleotide barcodes was added (Table A in S1 File) to the universal forward primer 338F (5'- ACTCCTACGGGAGGCAGCA -3') and the reverse primer 806R (5'- GGACTACHVGGGTWTCTAAT -3'). PCR products were quantified by an Agilent DNA 1000 Kit using an Agilent 2100 Bioanalyzer (Agilent Technologies, Santa Clara, CA) according to the manufacturer’s instructions. The amplification products were pooled in equimolar ratios with a final concentration of 100 nmol/L. The pools were sequenced by the Illumina MiSeq platform using barcoded primers in Shanghai Major-Bioscience Company.

### Quantification of predominant genera in pepper samples

The predominant bacteria in pepper peeling were detected by quantitative (q)-PCR, which was performed using an ABI Step-One detection system (Applied Biosystems). Based on the microbial abundance detected by high-throughput sequencing, we chose the following genera as target microbes for quantification: *Selenomonas*, *Prevotella*, *Clostridium*, *Tatumella*, *Weissella*, and *Enterobacter*. The primers used for detecting the abovementioned genera and the 20 μl PCR reaction mixtures were prepared as described previously [20, 21].

### The determination of the activity of acetyl xylan esterase and pectinesterase

The pepper samples for enzyme activities measurement were the same as meta 16S analysis, and they were collected in triplicate. The pepper peeling water in different time points were collected, and 10 mL of the water was centrifuged at 4°C and 12,000 g for 10 minutes, then the supernate was used for enzyme detection. The specific detection method was performed as previous study described. [22, 23]

### Bioinformatic and statistical analyses

Raw sequence data were trimmed to remove low-quality sequences, and the pair-end reads were merged by IDBA (v 1.0) software. Bioinformatic analyses were performed using QIIME (v1.6) [24] on the extracted high-quality sequences. Briefly, the sequences were aligned using PyNAST [25] and clustered under 100% sequence identity using UCLUST [26] to obtain the unique V3-V4 sequence set. After representative sequences were selected, the unique sequence set was classified into operational taxonomic units (OTUs) with a 97% threshold identity using UCLUST. ChimeraSlayer [27] was employed to remove any potentially chimeric sequences in the representative set of OTUs. The taxonomy of each OTU representative sequence was assigned using the Ribosomal Database Project [28] classifier with a minimum bootstrap threshold of 80%. OTUs that occurred only once or twice were discarded. A *de novo* taxonomic tree was constructed using a chimera-checked OTU representative set in FastTree [29] for downstream analyses, including alpha and beta diversity calculations. To evaluate alpha diversity, Shannon–Wiener and Simpson’s diversity indices, and the Chao1 and rarefaction estimators were calculated. UniFrac [30] metrics were calculated to evaluate beta diversity. Both weighted and unweighted calculations were performed prior to a principal coordinate analysis (PCoA).

All statistical analyses were performed using R software. PCoA and Procrustes analyses were performed in R using the ade4-package. Correlation core OTUs were calculated by Spearman’s rank correlation coefficient and visualized as a heatmap in R using the “pheatmap” package. Mantel test analyses were performed in R using the vegan package.

The sequence data reported in this paper have been deposited in the NCBI database (Accession Numbers: SRA: SRR2976395).

## Results

### Sequencing coverage and estimation of bacterial diversity

After 6 days retting, the peeling process have finished, and the peeled pepper was obtained. The microbiota composition of pepper pericarps at different peeling time points were examined using NGS. We generated a dataset consisting of 816,982 filtered high-quality and classifiable 16S rRNA gene sequences (42 samples) with an average of 19,443 sequences obtained for each individual. All sequences were clustered with representatives under conditions demanding 97% sequence identity. The number of OTUs varied between 124 and 323 (Table 1). Although the individual rarefaction curve failed to reach the saturation phase (Fig A in S1 File), the Shannon diversity estimates of the samples reached stable values (Fig A in S1 File). These results indicate that although new phylotypes would be expected with additional sequencing, most of the microbial diversity had already been captured.

### Changes in the structure and diversity of microbiota during pepper peeling

**Fig 1:**
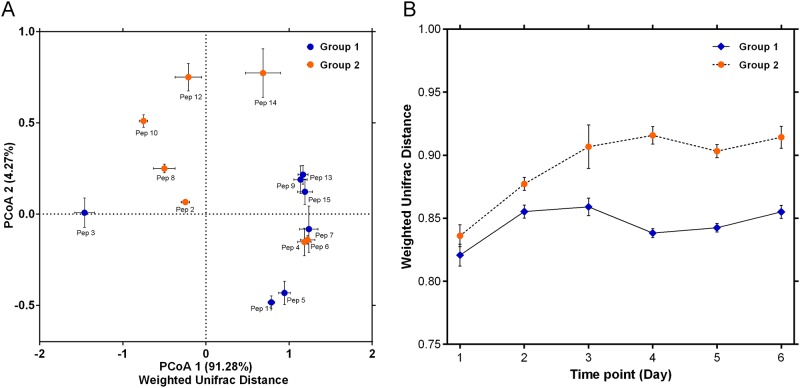
Changes in the microbial composition during pepper peeling. (A) A principal component (PCoA) score plot based on weighted UniFrac metrics for all samples. The orange points with error bars represent the pericarp microbial structure of pepper samples collected from Wanning city at different peeling time points and the points in blue represent pepper samples collected from Qionghai city. (B) The average weighted UniFrac distance between samples from day 0 and other days were calculated.

To explore the changes in the structure of pericarp microbiota during pepper peeling, PCoA based on weighted (Fig 1A) UniFrac distances was performed using the high-throughput sequencing data obtained from the pepper samples at different peeling time points. Within Fig 1a, orange points with error bars represent the pericarp microbial community structure of pepper samples collected from Wanning city at different peeling time points from day 0 (pre-peeling pericarp microbes on the pepper) to day 6. Similarly, the points in blue represent pepper samples collected from Qionghai city. The structure of the pericarp microbiota was shown to alter greatly during pepper peeling. Meanwhile, we also observed the pericarp microbial composition between the two sampling location was different (Fig 1A). To quantify these changes in microbial structure, we calculated the average weighted UniFrac distance between pepper samples on day 0 and other peeling samples from day 1 to day 6 (Fig 1B). For pericarp microbiota of samples in Qionghai pepper farm, the UniFrac distance to samples on day 0 peaked on day 3, then fall back on day 4 and increased again on day 6. The similar fluctuation also could be observed in samples in Wanning. The distance between samples on day 0 and other time points increased gradually, indicating that changes in microbial compositions intensified over time. The distance between samples on day 0 and other time points increased gradually, indicating that changes in microbial compositions intensified over time.

**Fig 2:**
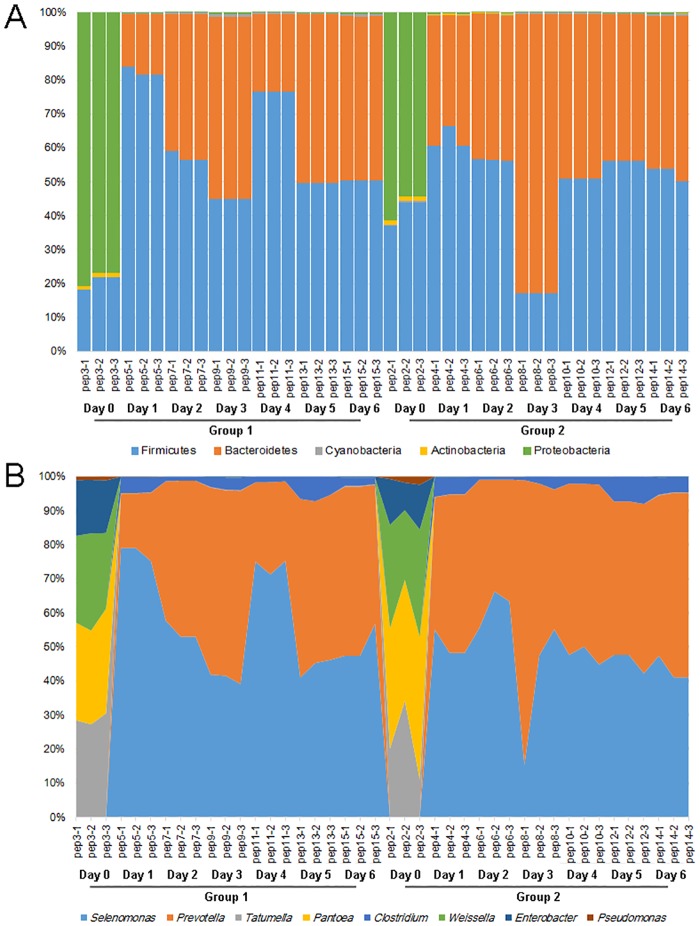
Average relative abundance of pericarp microbiota at the phylum (A) and genus (B) level during pepper peeling. The samples labeled pep3, 5, 7, 9, 11, 13 and 15 represented peeling samples on day 0 to day 6 collected in the pepper farm of Qionghai city. The samples labeled pep2, 4, 6, 8, 10, 12 and 14 represented peeling samples on day 0 to day 6 collected in the pepper farm of Wanning city.

After identifying an intrinsic difference in the pericarp microbiota composition during pepper peeling, we further examined the difference in specific bacteria at the phylum and genus level (Fig 2A and 2B). The abundance of pepper pericarp microbes from phyla Firmicutes and Bacteroidetes increased sharply while those from phylum Proteobacteria decreased sharply during pepper peeling compared with day 0. At the genus level, *Selenomonas*, *Prevotella*, and *Clostridium* increased notably in abundance and became the dominant microbes during pepper peeling.

### Core microbiota during pepper peeling

**Fig 3:**
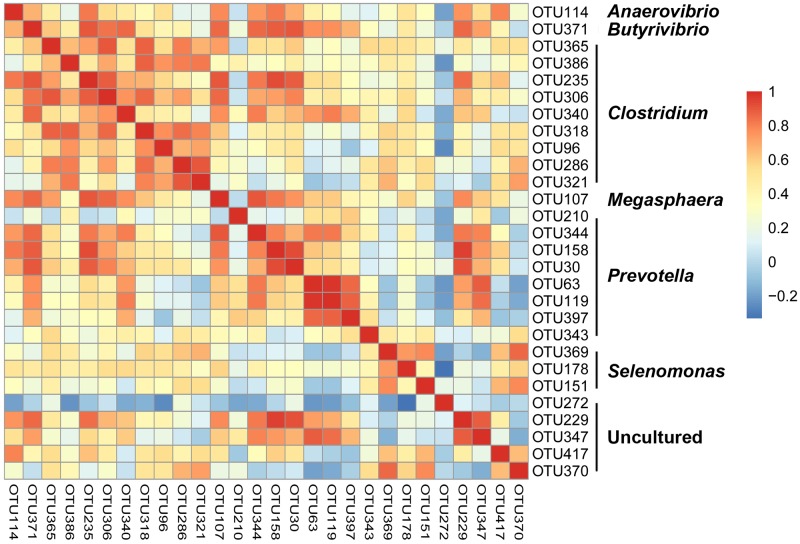
Core pericarp microbes were identified at the OTU level during peeling, and the correlation coefficient of the 28 core OTUs is shown as a heatmap.

A major interest in the present study is to determine whether a common core microbiota is shared between all pepper samples during peeling, because these microbes are likely to play a key role in the production of enzymes that promote pepper peel decomposition. Based on the OTU table, we calculated the occurrence frequency and the average abundance of each OTU. Then we identified 28 core OTU candidates (from a total of 423 OTUs identified in this study) (Fig 3), each with an average abundance of >0.1% in all samples. These core OTUs mainly belonged to the genera *Selenomonas*, *Prevotella*, *Megasphaera*, *Anaerovibrio*, *Butyrivibrio*, and *Clostridium*. Additionally, three of the 28 core OTU candidates, mainly belonging to the genus *Selenomonas*, were stably detected in every sample. Meanwhile, we calculated the correlation among the 28 core OTUs by Spearman’s correlation coefficient. A general negative correlation was found between *Selenomonas* represented OTUs and other genera represented OTUs, and a general positive correlation was found between *Clostridium* represented OTUs and other genera represented OTUs. Using genus-specific primers, we quantified the predominant microbiota in pepper peeling (Table 2). The amounts of of *Selenomonas*, *Prevotella*, *Clostridium*, *Tatumella*, *Weissella*, and *Enterobacter* genera were 7.62 ± 0.16, 7.55 ± 0.09, 6.41 ± 0.31, 5.35 ± 0.17, 5.27 ± 0.18 and 5.05 ± 0.22, respectively, in log-transformed 16S rDNA gene copy number per gram of sample. And the results matched their relative contribution from sequencing reads.

**Table 2:** Quantification of predominant genus during pepper peeling by q-PCR.

Genus	Relative contribution of sequencing reads (%)	Mean value of q-PCR (Log gene copy number)	Range of q-PCR values (Log gene copy number)
***Selenomonas***	41.74±9.84	7.62 (*n* = 42)	5.52–8.13
***Prevotella***	35.76±3.21	7.55 (*n* = 42)	5.17–7.86
***Clostridium***	2.57±1.17	6.41 (*n* = 42)	5.09–7.22
***Weissella***	1.58±0.41	5.35 (*n* = 42)	4.89–6.25
***Tatumella***	1.87±0.45	5.27 (*n* = 42)	3.85–6.37
***Enterobacter***	1.12±0.19	5.05 (*n* = 42)	4.24–5.49

### Changes in the functional features of pepper pericarp microbiota during peeling

**Fig 4:**
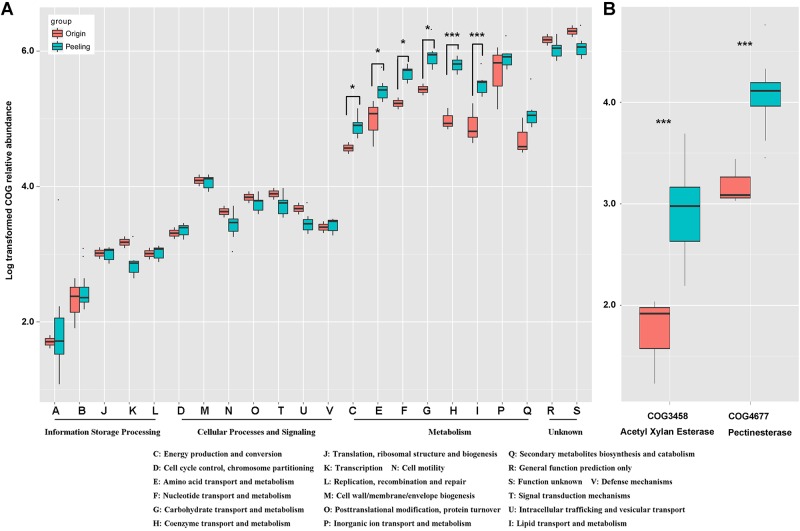
Functional features of the pericarp microbiota during peeling. The color in red represented the samples before peeling and the color in green represented the samples during peeling. (A) Comparison of functional broad headings between samples on day 0 and other time points. A: RNA processing and modification; J: Translation, ribosomal structure, and biogenesis; D: Cell cycle control, cell division, chromosome partitioning; M: Cell wall/membrane/envelope biogenesis; U: Intracellular trafficking, secretion, and vesicular transport; V: Defense mechanisms; H: Coenzyme transport and metabolism; P: Inorganic ion transport and metabolism; R: General function prediction only. (B) Comparison of COG distribution of acetyl xylan esterase and pectinesterase between pepper fruits and other peeling samples.

To better understand the key role of microbiota in pepper peeling, PICRUSt software (V 1.0) was used to predict 16S rRNA-based high-throughput sequencing data with the Cluster of Orthologous Groups (COG) database. After obtaining the COG profile, we correlated the microbial functional features with the important enzymes for pepper peeling. Compared with the microbial functional features on day 0, the functional broad heading related to metabolism increased in vigor during pepper peeling. The microbial functional features related to energy production and conversion, amino acid transport and metabolism, nucleotide transport and metabolism, carbohydrate transport and metabolism, coenzyme transport and metabolism, and lipid transport and metabolism increased significantly during peeling (Fig 4A). Additionally, at the higher resolution functional unit level, we observed a significant increase in COG3458 related to acetyl xylan esterase and COG4677 related to pectinesterase (Fig 4B). These two enzymes appear to play important roles in pepper peeling. To confirm the predicted functional genes related to pepper peeling, we detected the activity of acetyl xylan esterase and pectinesterase in different time points from day 0 to day 6 (Table 3). It can be observed the activity of acetyl xylan esterase kept increased from day 0 to day 6, and the activity of pectinesterase peaked around day 3, then fall back from day 4. Accordingly, after 6 days of peeling, the peeling process had finished. By using the Mantel test in R, we calculated the concordance between the microbial functional gene matrix and the enzyme activities matrix. Based on 999 permutations, we observed the mantel statistic r value was 0.6436, and the significance (*P* value) was 0.009. Accordingly, we confirmed the significant correlation between the predicted functional genes and microbial metabolic enzyme by statistical results.

**Table 3:** The activity of acetyl xylan esterase and pectinesterase during peeling.

Time points	Acetyl xylan esterase (U/mL)	Pectinesterase (*1000U/mL)
**Day 0 (*n* = 6)**	-	1.75±0.27
**Day 1 (*n* = 6)**	0.012±0.002	8.86±0.31
**Day 2 (*n* = 6)**	0.015±0.001	9.12±0.35
**Day 3 (*n* = 6)**	0.018±0.001	9.41±0.14
**Day 4 (*n* = 6)**	0.023±0.002	9.38±0.09
**Day 5 (*n* = 6)**	0.035±0.002	8.51±0.37
**Day 6 (*n* = 6)**	0.035±0.001	8.27±0.22

Note: “-”represented non-detected.

### Concordance of the microbial structure and functional features

To test the degree of consistency between the observed clustering patterns for organism structure and functional structure, Procrustes analysis was performed on the Principal Component Analysis (PCA) matrix of the genus-level organism profile and that of the COG profile (Fig B in S1 File). The results revealed a significant correlation between microbial taxonomy and functional feature (*P*<0.001, using 10,000 Monte Carlo label permutations). They also indicated that the pepper pericarp microbiota was closely associated with the production of various enzymes for pericarp decomposition during pepper peeling. This suggested that the progress of pepper peeling depends, to some extent, on the diversity of the pepper pericarp microbiota.

## Discussion

In the present study, we used a high-throughput sequencing approach to explore the core microbes and their functional genes related to pericarp degradation during pepper peeling. A total of 816,982 filtered high-quality reads were obtained with an average of 19,443 sequences for each sample. Most of the Shannon diversity and observed OTU curves had reached a plateau, indicating that the current number of sequencing reads was sufficient for bioinformatics analysis.

PCoA analysis based on the UniFrac distance revealed that the structure of the pericarp microbiota altered greatly during pepper peeling, and that the microbial genera *Selenomonas* and *Prevotella* played a key role in this process. The genus *Prevotella* is commonly isolated from the soil, rumen, and animal gut [31, 32]. It is composed of a wide array of carbohydrate-fermenting and acetate- and hydrogen-producing bacteria such as *Prevotella ruminicola* [33, 34]. The genus is particularly known to be able to digest and degrade fiber [35]. An example of the genus *Selenomonas* was first described in 1683 by Antonie Van Leeuwenhoek as a crescent-shaped bacterium from an oral sample [36]. Later, *Selenomonas* was isolated from rumen and soil samples [37]. *Selenomonas* species degrade complex polysaccharides associated with plant cell wall components, which have multiple carbon flow routes for carbohydrate catabolism and ATP generation [37]. Previous research also reported that some species of *Selenomonas* take part in the succinate–propionate pathway and ferment fiber, which is essential for energy production [38]. Together, this indicates that bacteria from *Prevotella* and *Selenomonas* genera are likely to be involved in pericarp fiber degradation during pepper peeling.

To explore the common core microbes and their functional genes related to peeling, we collected pepper samples from two pepper farms located in Qionghai and Wanning of Hainan province. Although we identified 28 core OTUs which mainly belonging to *Selenomonas*, *Prevotella*, *Megasphaera*, *Anaerovibrio* and *Clostridium* in each sample of the two sampling location above, even demonstrated their functional genes related to the enzymes of pepper peeling, we also observed the pericarp microbial composition between the two sampling location was different (Fig 1A). Geographically, the pepper farm in Qionghai area is located in the central region of the Hainan province, and it is surrounded by mountains, whereas the Wanning area is located in the southeast of the Hainan province, near the sea. Hence, the geographical features between these areas are significantly different. In fact, the composition of microbes in a microecosystem that originate from different geographical regions is influenced by many factors. The local environment factor play an important role in shaping the pepper pericarp microbial species distribution.

The pepper pericarp is mainly made up of pectin, which is a polysaccharide based on D-galacturonic acid polymers [39, 40]. During ripening, pectin is degraded by pectinases. In our analysis of the microbial functional features, we observed an increase in vigor in metabolism-related microbial functions during peeling. Of note, COG3458 associated with acetyl xylan esterase and COG4677 associated with pectinesterase significantly increased in number during the peeling process. Accordingly, we determined the activity of acetyl xylan esterase and pectinesterase, and it can be observed the activity of acetyl xylan esterase and pectinesterase kept increased during pepper peeling. Further analysis based on mantel test demonstrated the robust correlation between the microbial functional gene and the enzyme activities. Therefore, we confirmed the pepper pericarp microbiota is the source of peeling-related enzymes, and that core microbes (such as those of *Selenomonas* and *Prevotella* genera) with their functional genes play a key role in pepper peeling by biodegradation. This basic research will provide the theoretical guidance for the further development of pepper biodegradation. In future work, we will focus on the application of core microbial genera identified in the present study in pepper peeling.

## Supporting Information

S1 File Contains Table A, Figs A and B. (PDF)
