# Supplementary material for: Key Microbiota Identification Using Functional Gene Analysis during Pepper (Piper nigrum L.) Peeling
Source: PLoS One. 2016 Oct 21;11(10):e0165206. doi: 10.1371/journal.pone.0165206 (PMC5074590; doi:10.1371/journal.pone.0165206)
Supplement: S1 File — (PDF) [file pone.0165206.s001.pdf]

Key microbiota identification using functional gene analysis during pepper (*Piper nigrum* L.) peeling

Jiachao Zhang[1#], Qisong Hu[1#], Chuanbiao Xu[1], Sixin Liu[2]*, Congfa Li[1]*

[1]College of Food Science and Technology, Hainan University, Haikou 570228, P. R. China

[2] College of Materials and Chemical Engineering, Hainan University, Haikou 570228, P. R. China

[#]These authors contributed equally to this work.

* Corresponding author:

Congfa Li, College of Food Science and Technology, Hainan University, Haikou 570228, P. R. China Tel: 86-898-66193581, E-mail: congfa@vip.163.com

Sixin Liu, College of Materials and Chemical Engineering, Hainan University, Haikou 570228, P. R. China Tel: 86-898-66257032, E-mail: sixinliu@126.com

Table A Barcode used in present research

Group	Sample	Barcode	Group	Sample	Barcode
	Pep3-1	CCGTGC		Pep2-1	ACGTCG
	Pep5-1	GCGTGT		Pep4-1	GCGTCT
	Pep7-1	ACTACA		Pep6-1	ACGTGA
	Pep9-1	GCTACG		Pep8-1	CCTGGC
	Pep11-1	CCTACT		Pep10-1	GCTGGT
	Pep13-1	GCTAGA		Pep12-1	ACATCA
	Pep15-1	ACTAGC		Pep14-1	GTACCG
	Pep3-2	CCTAGT		Pep2-2	CCGTAT
	Pep5-2	GCTATC		Pep4-2	CAGAGA
**Group 1 Qionghai City**	Pep7-2	CGAGCA	**Group 2 Wanning City**	Pep6-2	GTAAGC
	Pep9-2	AGAGCG		Pep8-2	CATAGT
	Pep11-2	GGAGCT		Pep10-2	AAGTTC
	Pep13-2	AGAGGA		Pep12-2	ATTTCG
	Pep15-2	GGAGGT		Pep14-2	GATCCT
	Pep3-3	CGAGTC		Pep2-3	AGCAGA
	Pep5-3	GGAGTG		Pep4-3	GTCAGT
	Pep7-3	GGCTCG		Pep6-3	GTAGTC
	Pep9-3	AGCTCT		Pep8-3	CCTGTG
	Pep11-3	GGCTGA		Pep10-3	TCATCG
	Pep13-3	AGCTGC		Pep12-3	TAGCTCT
	Pep15-3	AGTCCA		Pep14-3	CCGTGA

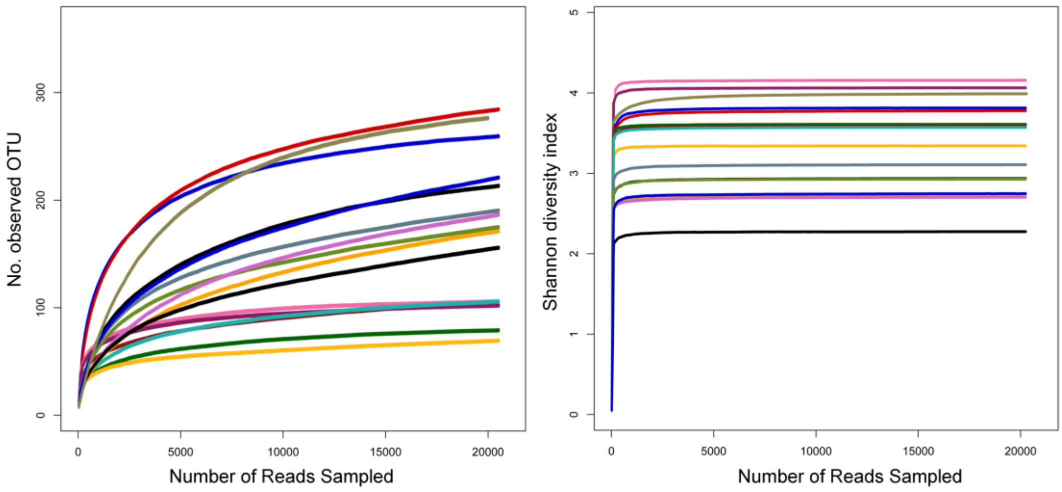

Fig. A Evaluation of α-Diversity. Shannon index and observed OTUs for all the samples were plotted. Each color represents the sample of an individual.

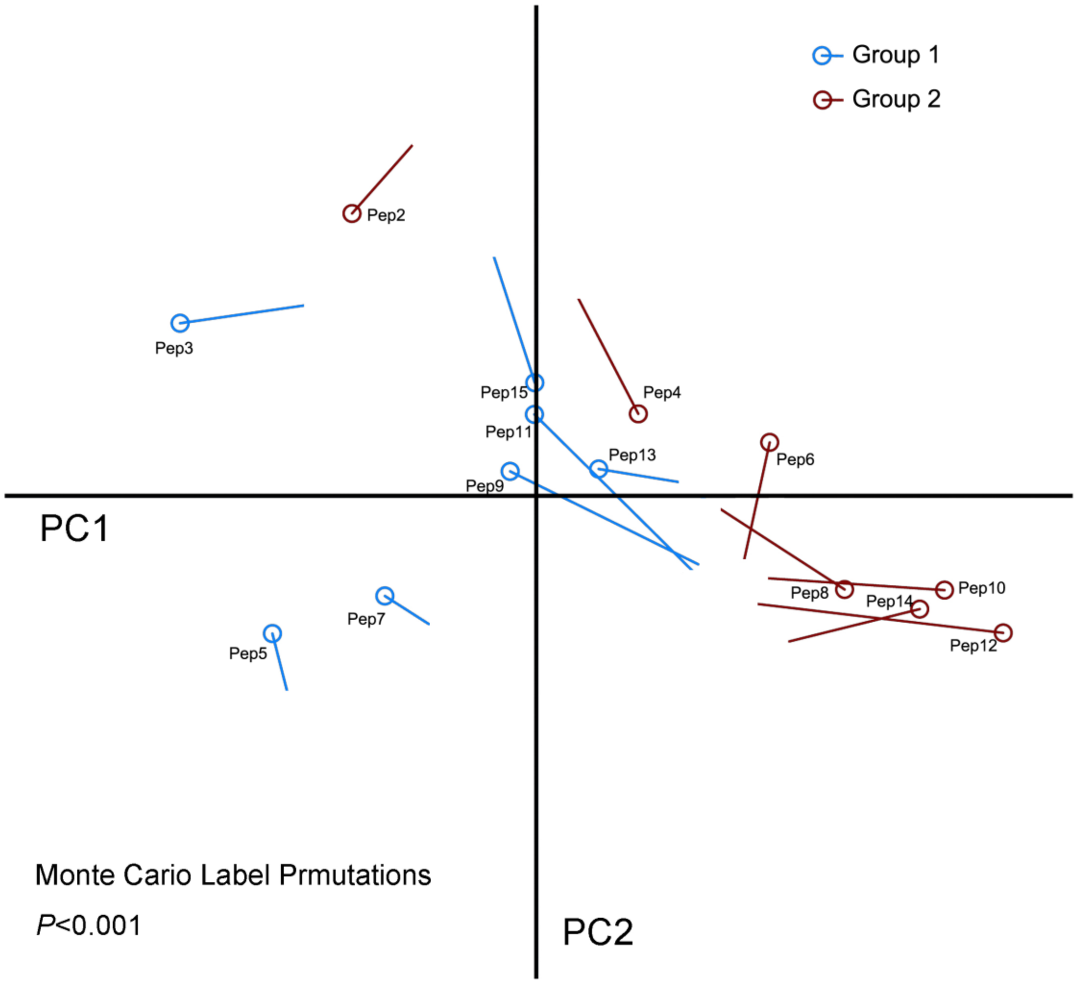

Fig. B Procrustes analysis revealed a strong correspondence between microbial taxonomy and function feature. The samples labeled pep3, 5, 7, 9, 11, 13 and 15 represented peeling samples on day 0 to day 6 collected in the pepper farm of Qionghai city. The samples labeled pep2, 4, 6, 8, 10, 12 and 14 represented peeling samples on day 0 to day 6 collected in the pepper farm of Wanning city. The cycle end of each line represented the 16S rRNA data for the sample, whereas the other end represented to the functional annotation of that particular sample. The fit of each Procrustes transformation over the first four dimensions was reported as the *P*-value by 10, 000 Monte Carlo label permutations.
